# Severe peach allergy in patients non-sensitized to Pru p 3

**DOI:** 10.1186/2045-7022-3-S3-P150

**Published:** 2013-07-25

**Authors:** MC Leoni, D Caimmi, AM Chiriac, MP Demoly, P Demoly

**Affiliations:** 1 Département de Pneumologie et Addictologie, Unité d’Allergologie INSERM U657, Hôpital Arnaud de Villeneuve, University Hospital of Montpellier, Montpellier, France

## Background

Allergy to Rosaceae is the fourth leading cause of food allergy in children in France. Typically, in Northern Europe, there is a prior sensitization to birch, through a PR-10 protein, while in Southern Europe the allergy is often preceded by a sensitization to a Lipid Transfer Protein (LTP). In Spain it is often recorded as a subsequent sensitization to apple (cross-reaction between Pru p 3 and Mal d 3). Beside, it has to be pointed out the frequent association between allergy to cypress and peach.

## Methods

We present two patients allergic to peach. The first one is a 23 year old female who experienced 4 episodes of facial angioedema, after ingestion of peach, and an episode of lingual angioedema after intake of raw apple. The second one is a 34 year old female allergic to cypress who referred an episode of anaphylaxis characterized by abdominalgia, facial angioedema, generalized urticaria, and dyspnea, after eating a peach. Both patients underwent a complete allergy work-up, from Skin Prick Tests (SPT) to Oral Provocation Test (OPT).

## Results

In the first patient, SPT were positive for mites, cypress, birch, peach and apple and prick by prick were positive to 4 different varieties of apple. Specific IgE were 53.2 kU/l for cypress, 0.65 kU/l for peach, 0.83 kU/l for apple and negative for peach recombinants rPru p 1, rPru p 3 and rPru p 4. An OPT to apple was positive at the end of the test, with facial urticaria. The OPT to peach was interrupted for an anaphylactic reaction including generalized urticaria and bronchospasm. In the second patient, SPTs were positive for mites, cypress and apple, and prick by prick were positive for apple. Specific IgE to peach were 2 kU/l, with negative results for rPru p 1, rPru p 3, and rPru p 4. The OPT to peach was positive at a dose of 32 grams, with abdominalgia, generalized urticaria and decrease of the FEV_1_ of 13% (440 ml).

## Conclusion

Peach allergy major determinant is Pru p 3, which is an LTP, but some patients are sensitized to Pru p 1 (PR 10) or Pru p 4 (profilin). Patients with severe reactions are usually sensitized to Pru p 3, but a co-sensitization to Pru p 1 and Pru p 4 seems to reduce the risk of important reactions. Nevertheless, none of the presented patients was sensitized to the allergenic components of peach that can be tested. It is therefore possible that there is a protein, not identified yet, that may be associated with severe reactions in patients allergic to peach.

## Disclosure of interest

None declared.

